# Associations of the Charlson comorbidity index with depression and mortality among the U.S. adults

**DOI:** 10.3389/fpubh.2024.1404270

**Published:** 2024-11-27

**Authors:** Ying-Zhao Wang, Chun Xue, Chao Ma, An-Bang Liu

**Affiliations:** ^1^Department of Neurology, Qianwei Hospital of Jilin Province, Changchun, China; ^2^Department of Gynecology and Obstetrics, The Second Affiliated Hospital of Naval Medical University, Shanghai, China; ^3^Department of Thoracic Surgery, Central Hospital Affiliated to Shandong First Medical University, Jinan, China; ^4^Shandong First Medical University & Shandong Academy of Medical Sciences, Jinan, China; ^5^Department of Cardiology, Central Hospital Affiliated to Shandong First Medical University, Jinan, China

**Keywords:** CCI, depression, mortality, chronic diseases, NHANES

## Abstract

**Background:**

Chronic comorbidities are often associated with higher risks of depression and mortality. This study aims to explore the relationships between the Charlson Comorbidity Index (CCI) and depression, and their combined effect on mortality.

**Methods:**

This study made use of data gathered in the National Health and Nutrition Examination Survey (NHANES) from 2007 to 2018, including a collective of 23,927 adult participants. According to CCI score distribution, CCI was categorized into three groups (T1 with CCI = 0; T2 with CCI = 1; T3 with CCI ≥ 2). In the CCI ≥ 2 group, patients may have two or more chronic diseases. Multivariable logistic regression models were employed to explore the relationship between CCI and depression. The study utilized the Cox proportional hazards model to investigate the association between CCI, the combination of CCI and depression, and all-cause mortality.

**Results:**

Our analysis revealed that after adjusting for potential confounders, a positive association was found between CCI and depression (OR = 1.25, 95% CI: 1.21, 1.29). Moreover, a greater CCI was found to be closely linked to higher mortality in individuals with depression (HR = 1.14, 95% CI 1.11, 1.18). Stratifying CCI into tertiles, higher tertiles of CCI (T2, T3 vs T1) also showed positive associations with depression and all-cause mortality. For patients with CCI ≥2 (T3) combined with depression, the risk of mortality was significantly elevated compared to those with CCI = 0 (T1) and non-depressed participants (HR = 2.01, 95% CI: 1.60, 2.52).

**Conclusion:**

The study findings demonstrate a positive correlation between CCI and the risk of depression, along with an association with increased all-cause mortality among depression patients. Hence, it is important to prioritize the clinical care of patients with a high CCI (≥2) and depression in order to lower the chances of mortality.

## Introduction

With the exacerbation of population aging, the incidence of chronic diseases is gradually increasing, rendering multimorbidity a burgeoning public health concern (1). Multimorbidity exacts a substantial toll on the global economy and healthcare systems (2). In 1984, the Charlson Comorbidity Index (CCI) was first used in a cohort study of 685 breast cancer patients to predict 1-year mortality (3). It stands as the preeminent tool for quantifying comorbidities and assessing individuals’ 10-year mortality risk (4). Previous research reports have demonstrated the utility of CCI in predicting the risk of mortality, disability, and even hospitalization (5).

The CCI covers a range of long-term illnesses such as cancer, heart failure, kidney disease, stroke, intensive care, liver disease, and diabetes, among others (6–12). Chronic illnesses like diabetes and heart diseases are slowly increasing in frequency (8, 12, 13). Nevertheless, these persistent illnesses also play a role in the development and advancement of specific mental health conditions.

Studies across different medical and psychiatric settings indicate a high prevalence of depression among diabetes mellitus (DM) patients (14). Depression, a common mood disorder (15), is prevalent among adult patients diagnosed with type 1 and type 2 DM (16, 17). Depression tends to persist in DM patients and is associated with increased disability, decreased quality of life (18), increased somatic symptoms (19), increased healthcare costs, and even increased risk of mortality (20). Similarly, studies have shown an increasing prevalence of depression among patients with chronic kidney disease (CKD), especially among those on long-term dialysis (7).

Depression is also associated with an elevated risk of physical health conditions. Depression complicates optimal management of cardiovascular disease (CVD) by worsening cardiovascular risk factors and decreasing adherence to healthy lifestyles and evidence-based medical treatments (21). The screening for depression is a useful and effective way to understand an individual’s CVD risk profile (22). Depressive symptoms are associated with poor walking performance in chronic obstructive pulmonary disease (COPD) patients, screening and management of depression may be one of the important aspects of care for COPD patients (23). Major depression is an independent risk factor for heart failure in HIV+ adults (15). Depression is linked to increased frailty and a higher risk of all-cause mortality (24). More chronic diseases were linked to higher psychological distress, especially in women (25). Patients with type 2 DM have higher depression and lower quality of life due to higher blood glucose and aging (26).

Chronic diseases like DM, CKD, COPD, and CVD often lead to psychological conditions, particularly depression, with the risk of psychological distress rising as the number of chronic diseases increases (21, 23, 25–27). Since both chronic disease and depression lead to increased poor prognosis (21, 24), It is important for patients with multiple chronic diseases to be screened for depression (21, 22).

Depression is prominent in people with multiple chronic diseases (28, 29). Patients with multimorbidity may not be able to respond effectively to disease risk factors, thereby increasing the risk of depression (30). Moreover, an increase in the number of chronic illnesses may be associated with an increased risk of depression, especially in women (25). The CCI serves as a robust predictor of short-term mortality due to venous thromboembolism (31), prognostic outcomes in older patients with cancer (32), and the likelihood of unplanned readmission within 30 days for patients aged >65 (33). CCI is also associated with dementia, polypharmacy, frailty, low walking speed and low muscle strength (34). Both multimorbidity or high CCI, and depression may lead to poor prognosis (4, 5, 24, 33, 35).

However, limited research exists on the relationship between CCI and depression, and how they together affect mortality. Our study aims to examine the correlation between CCI and individuals suffering from depression by analyzing data from the NHANES database, as well as to investigate the link between CCI, depression, and all-cause mortality.

## Methods

### Study population

NHANES uses a sophisticated sampling method (stratified, multistage, clustered probability sampling) to evaluate the health of the non-institutionalized civilian population in the United States, reflecting the entire country, carried out by NCHS. NHANES collects extensive data, including demographics, physical examination findings, laboratory results, and various disease-related questionnaire surveys. Approval for the survey was granted by the Institutional Review Board at the National Center for Health Statistics. Additionally, all participants provide written informed consent voluntarily before being enrolled in the study. For more information about NHANES, please visit: https://www.cdc.gov/nchs/nhanes/index.htm.

Our study included a total of 34,770 American adults aged 20 and above during the NHANES survey cycles from 2007 to 2018. After excluding pregnant women, individuals with missing depression data, relevant covariates, and those lost to follow-up, the final sample size was 23,927.

### Assessment of CCI

The CCI score, derived from the comorbidity items, is a sum score that can be used to assess the overall health status of participants (4). Our study’s CCI assessment focused on chronic conditions listed in NHANES medical conditions, excluding psychiatric status. Specific assessments of CCI disease items are detailed in Supplementary Table S1.

In NHANES, we defined diagnosed diseases using the Medical Conditions Questionnaire (MCQ). For questions such as “Have you ever been told by a doctor that you have disease?,” participants answering affirmatively were considered to have the respective disease. Consistent with the definition of CCI disease items, we assigned scores to reported diseases, while those not included in the items were assigned a value of zero (36), as detailed in Supplementary Table S2.

### Assessment of depression status

The 9-item Patient Health Questionnaire (PHQ-9) was utilized to evaluate symptoms of depression. The depression status was examined, with a cut-off point of 10 or higher on the PHQ-9 scoring criteria (sensitivity and specificity for depression were 88% each) (37).

### Follow up and endpoint

Participant survival status and reasons for death were determined by cross-referencing their information with the National Death Index public records up to December 31, 2019. For details, please see the official website.^1^ The median duration of observation for individuals without depression was 82 months (interquartile range 50–119), while for those with depression, it was 83 months (interquartile range 51–121), for the overall participants is 82 (interquartile range 50–119) months.

### Covariate

We selected covariates based on considerations of common demographic parameters and clinical experience. The variables analyzed in the study included age, gender, ethnicity, BMI, PIR, marital status, educational background, smoking habits, drinking habits, exercise, cholesterol levels, and hypertension.

Participants provided self-reported information on their age (≥20), gender (male and female), and race (Non-hisp. White, Non-hisp. Black, Mexican American, Other Race), marital status (Married/living with partner, Single/divorced/widowed), education level (High school or lower, College or higher), poverty to income ratio (PIR), smoking habits (Never, Former, Current), and alcohol intake (Never, Former, Light, Moderate, Heavy). The calculation of body mass index (BMI) involved dividing the weight (in kilograms) by the square of the height (in meters). Physical activity levels were determined using a questionnaire focusing on leisure-time activities, with individuals who engaged in at least 300 min per week of moderate activity, 150 min per week of vigorous activity, or a combination of both considered to be active. Total cholesterol was measured via plasma or serum assays. Hypertension was characterized by having a systolic blood pressure (SBP) of at least 140 mmHg, a diastolic blood pressure (DBP) of at least 90 mmHg, or taking antihypertensive medication.

### Statistical analysis

This study examined the characteristics of American adults based on depression status. Sampling weights were utilized in all analyses to ensure unbiased estimates in the complex NHANES design, with Taylor series (linearization) used for estimating standard errors (SE). Continuous variables were reported as mean (±SE) and compared using *t*-tests or Wilcoxon rank-sum tests depending on the normality test results. Categorical variables were displayed as frequencies with weighted percentages and then analyzed using chi-square tests.

Logistic regression was used to determine the odds ratios (ORs) and 95% confidence intervals (CIs) for the association between CCI and depression, with adjustments for covariates. Cox proportional hazards models were used to determine the hazard ratios (HRs) and their 95% CIs in order to investigate the relationship between combined CCI, tertiles of CCI, and depression with all-cause mortality. Based on CCI definition, NHANES medical conditions, and CCI score distribution, CCI was divided into tertiles (T1 = 0, T2 = 1, T3 ≥ 2). The crude model was univariable analysis, while Model I was adjusted for age, gender, race, and BMI. Model II was additionally adjusted for all variables in Model I as well as additional covariates such as PIR, education, marital status, smoking, alcohol consumption, physical activity, hypertension, and TC. Due to the overdispersion between depressed and non-depressed groups, we used depression count by negative binomial model for sensitivity analyses (Supplementary Table S3). In addition, considering the large population excluded from the study, we also performed sensitivity analyses for excluded adult participants with CCI assessment (*N* = 10,840). Analyses included frequency distributions of CCI in excluded participants (Supplementary Figure S1), univariable logistic regression on association of CCI with depression (Supplementary Table S4), and univariable Cox proportional hazards regression on association of CCI with mortality (Supplementary Table S5).

To detect possible non-linear relationships, we applied restricted cubic spline (RCS) with 4 knots (5, 35, 65, and 95%) set to explore the relationship of CCI (continuous variable) with depression and mortality. Use Kaplan–Meier method to estimate the tertiles of CCI, as well as CCI and depression status with cumulative survival probability, and compare them using log-rank test. A *p*-value less than 0.05, indicating statistical significance. All analyses were conducted using R (version 4.2.1).

## Results

### Study participants and baseline characteristics

**Figure 1:**
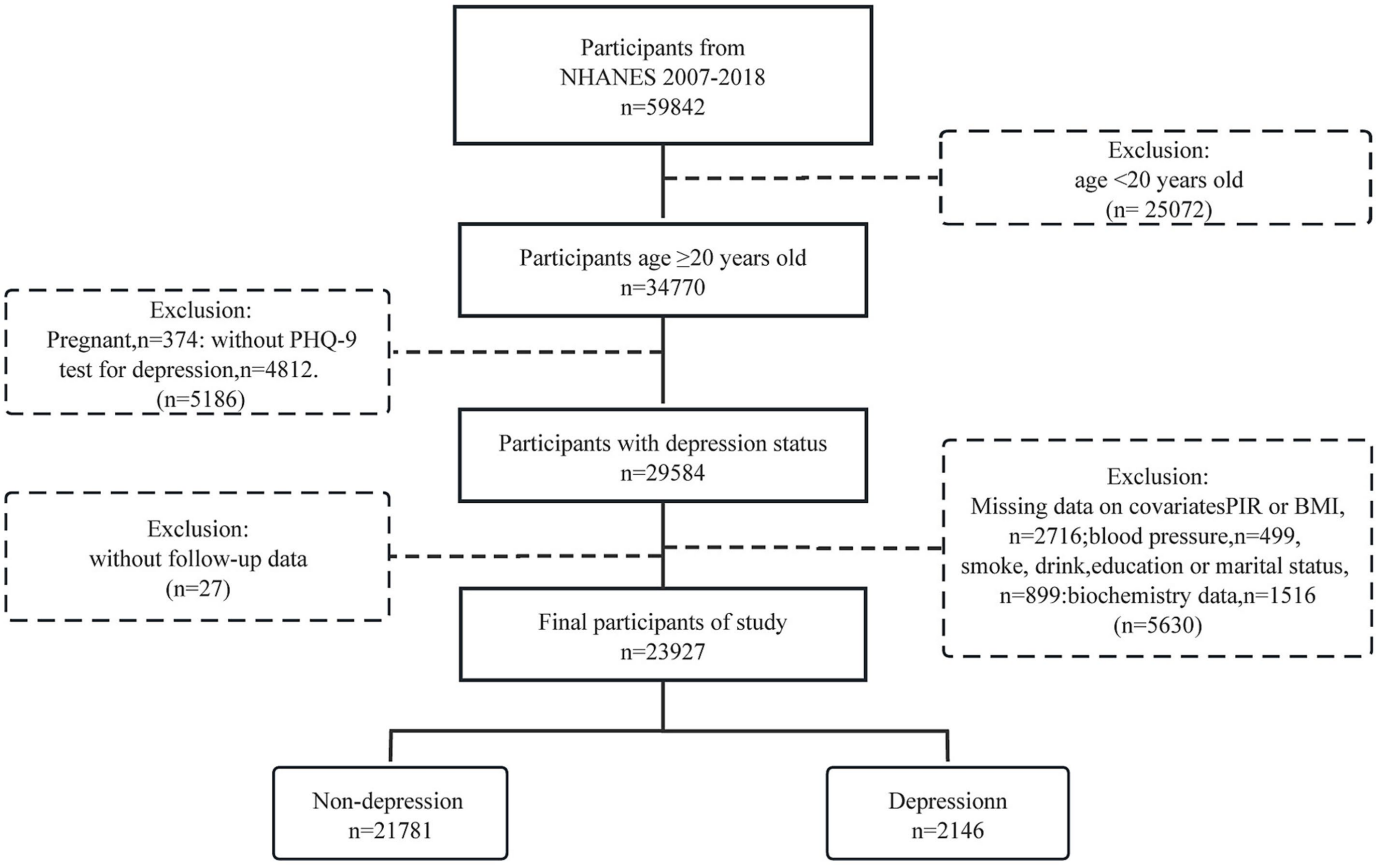
Participants selection of the study.

The study involved 23,927 American individuals aged 20 years and above, among whom 2,146 were identified as having depression (Figure 1 and Table 1). Participants had an average age of 47.36 years, with males making up 49.52% of the group (*n* = 11,956), and an average CCI score (continuous) of 0.96. Individuals with depression were significantly more likely to be female, divorced, single, or widowed, have a high BMI, high DBP, lower education level, current smokers, and high total cholesterol levels compared to those without depression.

**Table 1:** Baseline characteristics of participants stratified by depression status.

Characteristics	Overall	Non-depression PHQ9 < 10	Depression PHQ9 ≥ 10	*P*-value
Number of participants	23,927	21,781	2,146	
Age	47.36(0.26)	47.44(0.28)	46.38(0.47)	0.05
Sex				<0.0001
Male	11,956(49.52)	11,164(50.63)	792(36.53)	
Female	11,971(50.48)	10,617(49.37)	1,354(63.47)	
Race/Ethnicity				<0.001
Non-Hisp. Black	4,785(10.05)	4,347(9.86)	438(12.35)	
Non-Hisp. White	10,534(69.25)	9,585(69.58)	949(65.38)	
Mexican American	3,543(8.15)	3,237(8.21)	306(7.47)	
Other Race	5,065(12.55)	4,612(12.36)	453(14.80)	
BMI (kg/m^2^)	29.14(0.09)	29.00(0.09)	30.78(0.23)	<0.0001
SBP (mmHg)	121.70(0.20)	121.75(0.20)	121.14(0.51)	0.24
DBP (mmHg)	70.90(0.21)	70.88(0.22)	71.17(0.34)	0.41
PIR	3.05(0.04)	3.12(0.04)	2.15(0.07)	<0.0001
Marital status				<0.0001
Married, living with partner	14,309(63.79)	13,353(65.16)	956(47.68)	
Single, divorced, or widowed	9,618(36.21)	8,428(34.84)	1,190(52.32)	
Education				<0.0001
High school or below	10,842(37.09)	9,599(35.95)	1,243(50.47)	
College or above	13,085(62.91)	12,182(64.05)	903(49.53)	
Smoke				<0.0001
Never	13,158(55.42)	12,305(56.89)	853(38.15)	
Former	5,845(25.10)	5,358(25.29)	487(22.81)	
Now	4,924(19.48)	4,118(17.82)	806(39.04)	
Alcohol drinking				<0.0001
Never	3,245(10.23)	2,996(10.38)	249(8.45)	
Former	3,710(12.61)	3,248(12.05)	462(19.22)	
Mild	8,221(37.42)	7,670(38.22)	551(27.99)	
Moderate	3,789(17.94)	3,453(17.96)	336(17.72)	
Heavy	4,962(21.80)	4,414(21.39)	548(26.62)	
Physical activity				<0.0001
Active	5,388(25.85)	5,144(26.91)	244(13.43)	
Inactive	18,539(74.15)	16,637(73.09)	1902(86.57)	
Hypertension	10,191(37.78)	9,109(37.00)	1,082(46.94)	<0.0001
TC (mg/dL)	194.11(0.53)	193.96(0.52)	195.83(1.35)	0.15
CCI	0.96(0.02)	0.91(0.02)	1.52(0.04)	<0.0001
All-cause mortality	2,176(6.45)	1937(6.26)	239(8.73)	<0.001

PHQ9, Patient Health Questionnaire; BMI, body mass index; SBP, systolic blood pressure; DBP, diastolic blood pressure; PIR, poverty-to-income ratio; TC, total cholesterol; CCI, Charlson Comorbidity Index.

### The correlation of CCI with depression

Logistic regression analysis was used to investigate the correlation between CCI score and depression, with adjustments for covariates as shown in Table 2. Upon analyzing the CCI score as a continuous factor, the adjusted OR for depression was determined to be 1.25 (95% CI 1.21, 1.29). In the analysis of CCI scores grouped into tertiles, individuals in the T2 and T3 categories had a notably higher odds of experiencing depression when compared to those in the lowest T1 category in the basic model, indicating a positive correlation between depression risk and higher CCI score tertiles T2 and T3.This pattern persisted after adjusting for age, gender, race, and BMI in Model 1.In the second model, even after accounting for all factors in the first model and additional covariates, the correlation between CCI score tertiles T2 and T3 in comparison to T1 persisted, showing a substantial increase in the likelihood of depression at T3 compared to T1, with an odds ratio of 2.26 and a 95% CI of 1.98–2.57.

**Table 2:** Odds ratio (95% confidence intervals) for CCI associated with depression.

	Odds ratio (95% CI)		
	Crude model	Model I	Model II
CCI	1.24(1.21, 1.28)	1.33(1.29, 1.37)	1.25(1.21, 1.29)
Tertiles of CCI			
T1, CCI = 0	Ref	Ref	Ref
T2, CCI = 1	1.60(1.37, 1.86)	1.93(1.63, 2.28)	1.57(1.33, 1.86)
T3, CCI = [2, 12]	2.24(1.99, 2.53)	2.90(2.56, 3.29)	2.26(1.98, 2.57)

Crude model was univariable logistic regression model. Model I adjusted for age, sex, ethnicity and BMI. Model II adjusted for age, sex, ethnicity, BMI, PIR, education, marital status, smoke, alcohol drinking, physical activity, hypertension, and TC.

In negative binomial model, holding other variables constant, for every 1 increase in CCI, the expected change in depression was 1.21 (RR = 1.21, *p* < 0.0001). In the logistic regression part, the expected change in depression was 1.25 (OR = 1.25, *p* < 0.0001) for every 1 increase in CCI (Supplementary Table S3).

**Figure 2:**
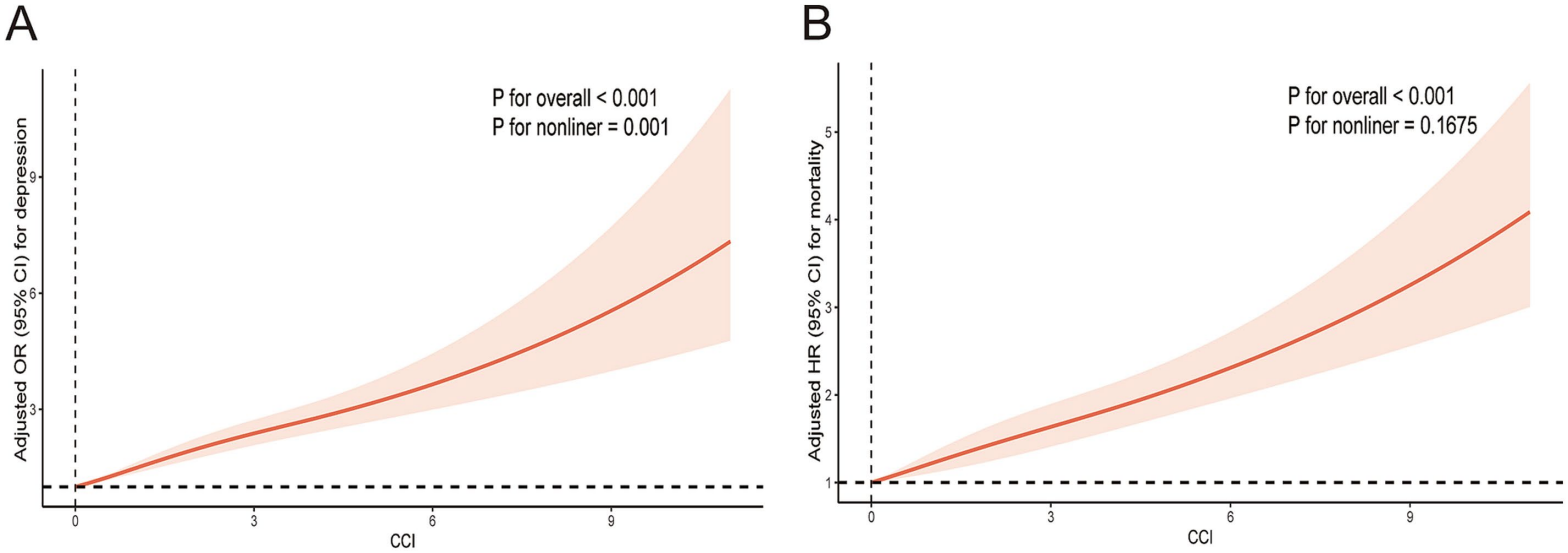
Restricted cubic spline plotting of CCI in relation to depression **(A)** and mortality **(B)**. The results adjusted for age, sex, ethnicity, BMI, PIR, education, marital status, smoke, alcohol drinking, physical activity, hypertension, and TC.

Moreover, CCI was non-linearly and positively associated with depression (*P* for overall <0.001, *P* for non-linear =0.001) (Figure 2A).

### The correlation between CCI score and all-cause mortality

**Figure 3:**
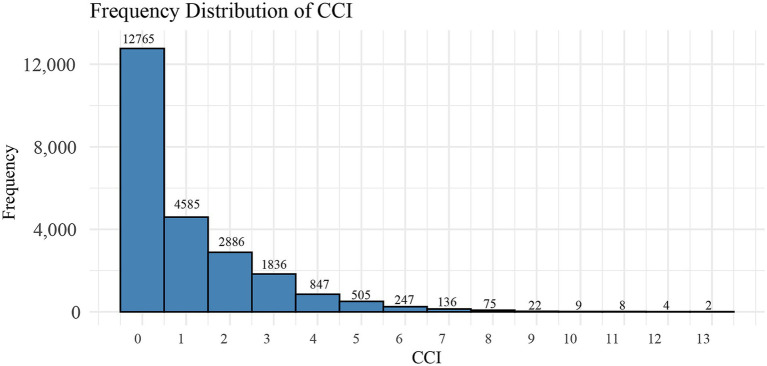
Distribution of Carlson comorbidity index among participants in this study.

**Figure 4:**
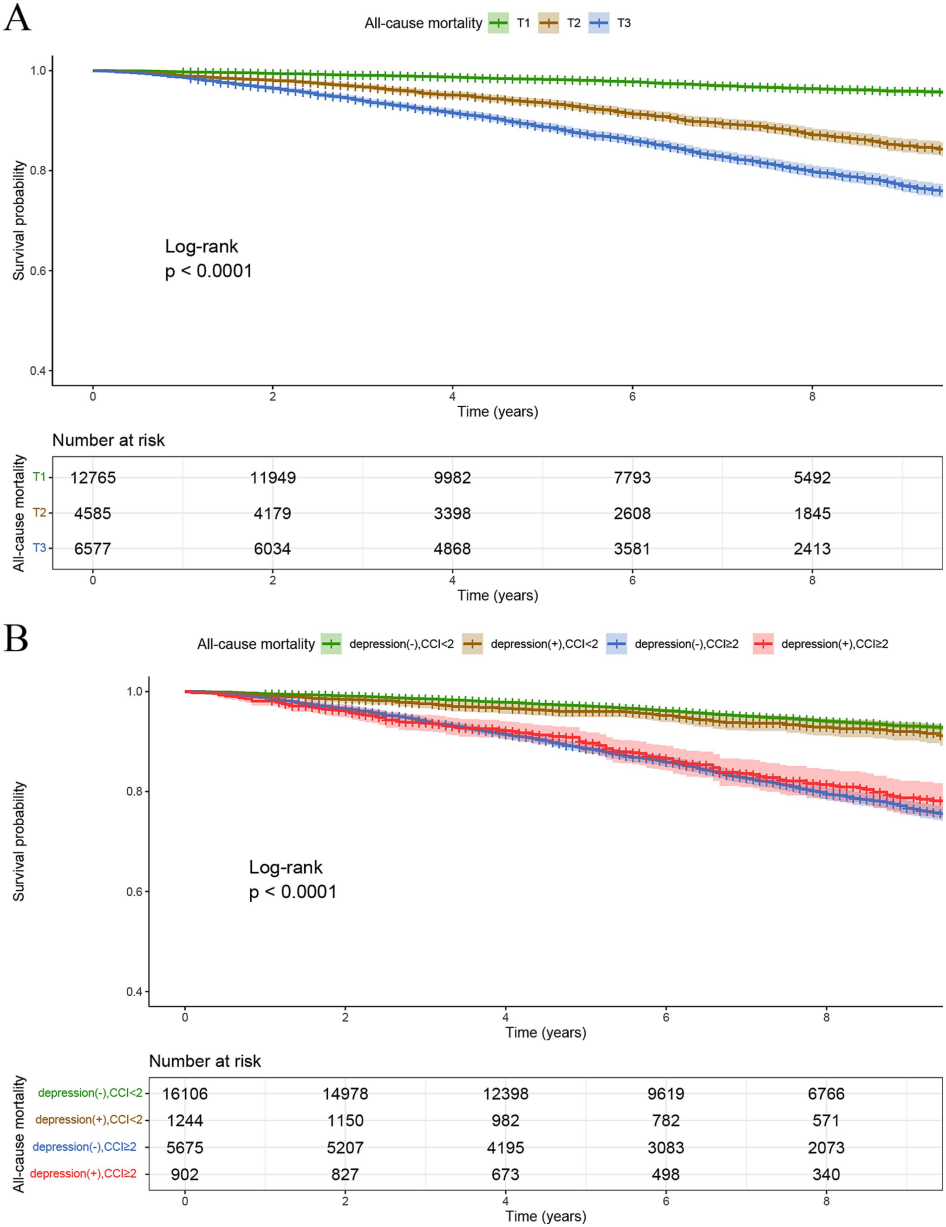
Kaplan–Meier survival curves for all-cause mortality stratified by tertiles of CCI **(A)**, and CCI/depression group **(B)**.

All participants were followed up until December 31, 2019. The median follow-up duration was 82 (50, 119) months for the non-depression group and 83 (51, 121) months for the depression group. In the frequency distribution of CCI scores (Figure 3), a gradual decrease in the number of participants was observed with increasing CCI scores. When the CCI was 0, the number of participant (*n* = 12,765) were notably higher than at other CCI scores. Analysis of Kaplan–Meier survival curves showed a notable link between elevated CCI scores and higher all-cause mortality in the entire study population (*P*-log rank <0.001) (Figure 4A).

Kaplan–Meier survival curve analysis indicated that the survival rates of those without depression and with CCI < 2 and those with depression and CCI < 2 were notably higher than those without depression and with CCI ≥ 2, and those with depression and CCI ≥ 2. This indicates that elevated CCI values are linked to higher rates of all-cause mortality. Additionally, the survival probability of individuals without depression and with CCI < 2 were significantly higher than those with depression and CCI < 2, indicating that depression similarly contributes to elevated all-cause mortality. Furthermore, among the other two groups (CCI ≥2), individuals with depression experienced higher all-cause mortality than those without depression during early survival days (Figure 4B).

Moreover, CCI was linearly and positively associated with mortality (*P* for overall <0.001, *P* for non-linear =0.1675) (Figure 2B).

In the three models, namely the crude model, Model 1, and Model 2, Model 2 was adjusted for all variables and additional covariates from Model 1, showing consistent results with Model 1. The HRs for mortality in the higher CCI tertiles (T2, T3) were consistently greater than those in the lowest CCI tertile (T1). For instance, in Model 2, the HR for the T3 tertile was 1.73 (1.50, 2.00), significantly higher than that for the T1 tertile of CCI. In the combined grouping of depression and CCI tertiles, participants were primarily divided into six groups based on depression status and CCI scores: non-depression with CCI = 0 (T1), depression with CCI =0 (T1), non-depression with CCI =1 (T2), depression with CCI =1 (T2), non-depression with CCI ≥2 (T3), and depression with CCI ≥2 (T3). In Model 2, it showed a significant rise in all-cause mortality risk for participants with depression compared to those without depression, as well as a noticeable increase in all-cause mortality with higher CCI tertiles, with HRs and 95% CIs of 1.28 (0.76, 2.14), 1.28 (1.06, 1.55), 1.57 (1.08, 2.29), 1.72 (1.48, 1.99), and 2.01 (1.60, 2.52) for the 5 groups respectively, compared to group of non-depression with CCI = 0 (T1). It can be assumed that depression can synergize with CCI to exacerbate poor prognosis (Table 3).

**Table 3:** Associations of CCI, tertiles of CCI, and CCI/depression group with mortality by Cox proportional hazard regression models.

	Hazard ratio (95% confidence intervals)		
	Crude model	Model I	Model II
CCI	1.46(1.43, 1.49)	1.19(1.15, 1.22)	1.14(1.11, 1.18)
Tertiles of CCI			
T1, CCI = 0	Ref	Ref	Ref
T2, CCI = 1	3.86(3.26, 4.56)	1.44(1.21, 1.72)	1.29(1.08, 1.54)
T3, CCI = [2, 12]	6.62(5.63, 7.78)	2.04(1.75, 2.38)	1.73(1.50, 2.00)
CCI/depression group			
T1, depression (−)	Ref	Ref	Ref
T1, depression (+)	1.20(0.73, 1.99)	1.93(1.15, 3.24)	1.28(0.76, 2.14)
T2, depression (−)	3.86(3.24, 4.59)	1.41(1.17, 1.69)	1.28(1.06, 1.55)
T2, depression (+)	4.30(3.05, 6.08)	2.59(1.81, 3.70)	1.57(1.08, 2.29)
T3, depression (−)	6.65(5.62, 7.88)	1.97(1.68, 2.31)	1.72(1.48, 1.99)
T3, depression (+)	6.99(5.62, 8.68)	3.41(2.73, 4.24)	2.01(1.60, 2.52)

Crude model was univariable Cox proportional hazard regression model. Model I adjusted for age, sex, ethnicity and BMI. Model II adjusted for age, sex, ethnicity, BMI, PIR, education, marital status, smoke, alcohol drinking, physical activity, hypertension, and TC.

### Sensitivity analyses for excluded adult participants

A large number of adult individuals were excluded from the study (34,770–23,927 = 10,843, Figure 1), of which *N* = 10,840 had CCI assessments. We plotted the frequency distribution of CCI for the excluded 10,840 samples and found that the distribution was similar to the 23,927 included in our study (Supplementary Figure S1).

In addition, we explored the association of CCI with depression and death in excluded participants. The univariable logistic regression analysis showed that each 1-score increase in CCI was significantly associated with a 26% increase in the risk of depression. CCI tertiles also showed a 2.91-fold increased risk of depression for CCI ≥ 2 (T3) versus CCI = 0 (T1), with an OR 95% CI of 2.91 (2.40, 3.53) (Supplementary Table S4). The univariable Cox proportional hazards regression indicated that a 1-score increase in CCI was associated with a significant 41% increase in the risk of all-cause mortality. It also showed 5.95 times increased risk of death for patients with CCI ≥ 2 (T3) relative to CCI = 0 (T1), with a HR 95% CI of 5.95 (5.20, 6.81). When patients of CCI ≥ 2 with depression, the risk of mortality was 7.23 times higher than that of CCI = 0 without depression, with an HR 95% CI of 7.23 (5.22, 10.02) (Supplementary Table S5).

In summary, we performed sensitivity analyses on excluded adult participants and found results similar with those of the included study population.

## Discussion

Our study used information from NHANES covering the years 2007 to 2018 to explore the connection between CCI scores and the likelihood of depression, as well as the correlation between CCI scores and all-cause mortality in people with and without depression. The findings indicate a positive correlation between CCI scores and the prevalence of depression. Moreover, individuals with higher CCI scores (CCI ≥2) exhibit elevated all-cause mortality among those with depression. Our results underscore the importance of considering chronic disease comorbidity burden in assessing the risk and outcomes of depression.

The CCI is essential in clinical practice, commonly used to evaluate patients’ well-being and forecast their outcomes. One study demonstrated that CCI serves as a reliable tool aiding clinicians in assessing patients’ overall health status and predicting the risk of adverse events occurrence (3). The connection between different chronic diseases and depression has received considerable focus, such as hypertension, DM, heart disease, COPD, and more. These chronic diseases not only affect patients’ physical health but may also have negative implications for their mental well-being, increasing the risk of depression (38–41). A study found that hypertensive patients are more likely to experience depression than those without hypertension (38). Another study involving heart failure patients showed a higher likelihood of depression compared to those with normal cardiac function (39). Furthermore, conditions like COPD and heart failure are closely associated with the occurrence of depression (40, 41). Nevertheless, there is a lack of extensive research on the correlation between CCI scores and depression. The CCI serves as a composite score for a variety of chronic diseases, so we systematically assessed the relationship between CCI scores and depression. The findings indicate a positive correlation between CCI scores and the depression.

Depression, a common emotional condition, is defined by long-lasting feelings of unhappiness or a lack of enjoyment, frequently leading to difficulties in daily functioning (15, 42, 43). Globally, depression contributes to increasing disability and years of life lost annually even in young people (44). In the United States, the prevalence of depression is around 5 to 10%, reaching up to 40 to 50% in certain healthcare or specialized settings (45). However, only half of depression sufferers receive treatment (46). Furthermore, among patients with comorbid conditions such as DM or heart disease, treatment rates are even lower, contributing to higher mortality (17, 47).

An increase in the number of chronic diseases is associated with an increased risk of psychological distress, and women are more likely to experience psychological distress (25). Depression is prominent in people with multiple chronic diseases (28, 29). On one hand, patients with chronic diseases often experience depression, sadness, and anxiety due to the ongoing effort required to manage their diseases and integrate them into daily life (48). On the other hand, Patients with multimorbidity often face depression that hinders daily activities and self-management. This also can make them less responsive to disease risk factors, potentially increasing their depression risk (30, 49).

Depression in hypertensive patients was associated with poor drug compliance, and this association increased with the severity of depression. Socioeconomic/demographic factors have independent effects on drug adherence, including gender, age, BMI, ethnicity, marital status, and health insurance (50). Improved adherence to antidepressants and antihypertensive medications improved depression outcomes and blood pressure control was observed in primary care patients (51). Depressive mood is an important factor contributing to low drug compliance in hemodialysis or kidney transplant recipients with end-stage renal disease (52). There is a strong association between depression and treatment non-compliance, and identifying sudden non-compliance may be a screening tool for depression (53). Depression exacerbates cardiovascular risk factors and reduces adherence to healthy lifestyle and evidence-based medical treatment, thereby complicating optimal management of CVD (21).

Our research results showed that patients with comorbid depression had a higher risk of all-cause mortality when their CCI scores were 2 or higher. Recent research has found that higher CCI scores are linked to poorer treatment adherence in patients with depression, indicating that individuals with depression tend to use fewer medical services compared to those without depression, even with similar CCI scores (54). This corroborates previous findings, as evidenced by a study involving 367 DM patients from two HMO primary care clinics in Washington, where patients with higher depressive symptoms were often less adherent to medication regimens (55). Similarly, in a 12-month longitudinal study of hypertensive patients, those with mild depression or anxiety showed inadequate adherence to antihypertensive treatment (56). Moreover, studies have demonstrated that depression adversely affects treatment adherence in chronic disease management (57). The mortality is higher in breast cancer or lung cancer patients with comorbid depression (58, 59). Adverse outcomes in cardiovascular disease patients such as coronary artery disease or heart failure are also increased when combined with depression (60, 61). Our research, in line with prior results, shows that individuals with higher CCI scores (CCI ≥ 2) have a greater likelihood of experiencing depression, and the risk of mortality rises for those with depression and high CCI scores. In summary, patients with chronic diseases and comorbid depression exhibit lower treatment adherence, implying a close association between high CCI values combined with depression and increased all-cause mortality.

Our study has several strengths. The study addresses the gap in research on the relationship between CCI, depression risk, and all-cause mortality, which has been limited. Previous studies were mostly limited to the association of single disease with depression or prognosis, and lack of knowledge gaps on the integrated score of various chronic diseases and depression and prognosis. CCI is appropriate tool to evaluate chronic disease. Notably, older populations often have multiple chronic conditions, such as DM, CVD, COPD, and depression, which can be improved through integrated care, increased use of mental health services, reduced depression, and improved health function-related status (62). Clinical practice encountered a variety of chronic disease population can be comprehensive screening of depression and assessment of prognosis risk. Secondly, NHANES implements rigorous quality assurance procedures and has a large representative sample, ensuring the validity of the data. Furthermore, we accounted for pertinent variables in order to more accurately assess the association between CCI and the likelihood of experiencing depression and all-cause mortality.

Although our study is novel and comprehensive, it has some limitations. Firstly, it is an observational study, limiting our ability to draw causal inferences. Moreover, some potential complex confounders that cannot be fully controlled may lead to certain biases in the results. Although we adjusted for most of the relevant confounding factors, residual or unknown confounding factors (e.g., differences in chronic disease drug use or dietary factors) could not be excluded. Moreover, the reliance on self-reports for most chronic diseases in NHANES may skew the CCI scores. Furthermore, according to CCI definition and NHANES medical condition questionnaire evaluation, CCI scores lack individual diseases with scores of 3 or above in NHANES. For diseases with CCI scores of 3 or above, for instance, disease like moderate or severe liver disease (scored 3) and AIDS/HIV (scored 6), NHANES lacks relevant information and could not be confirmed. Nevertheless, this does not diminish our comprehension of the connection between CCI and the likelihood of depression and mortality, offering fresh perspectives and hints for predicting the outcomes of individuals with chronic illnesses coexisting with depression.

## Conclusion

Our study found that elevated CCI scores were linked to higher chances of experiencing depression and mortality. In individuals diagnosed with depression, having a CCI score of 2 or higher was linked to a higher risk of mortality when compared to those without any comorbidities. These findings demonstrate the predictive value of CCI scores for depression and mortality and provide valuable insights into clinical practice in patients with multimorbidity of chronic disease and/or depression.

## Data Availability

The original contributions presented in the study are included in the article/Supplementary material, further inquiries can be directed to the corresponding authors.

## Footnotes

^1^ https://ftp.cdc.gov/pub/Health_Statistics/NCHS/datalinkage/linked_mortality/
