# Supplementary material for: Associations of the Charlson comorbidity index with depression and mortality among the U.S. adults
Source: Front Public Health. 2024 Nov 27;12:1404270. doi: 10.3389/fpubh.2024.1404270 (PMC11632622; doi:10.3389/fpubh.2024.1404270)
Supplement: Supplementary file 1 [file Table_1.docx]

**Supplementary materials**

**Supplementary Table S1**. Carlson Comorbidity Index (CCI) scores.

**Supplementary Table S2.** Carlson Comorbidity Index (CCI) scores evaluation from respondent’s comprehensive health status in enrolled participants of NHANES 2007-2018.

**Supplementary Table S3**. Predictors of depression among US adults

**Supplementary Table S4**. Associations of CCI, and tertiles of CCI with depression in excluded adult participants with PHQ-9 test.

**Supplementary Table S5**. Associations of CCI, tertiles of CCI, and CCI / depression group with mortality in excluded adult participants with follow up data.

**Supplementary Figure S1.** Distribution of Carlson comorbidity index (CCI) in excluded adult participants with CCI evaluation (N =10840).

**Supplementary Table S1**. Carlson Comorbidity Index (CCI) scores.

| Weighted Score | Conditions |
| --- | --- |
| 1 |  |
|  | Myocardial infarction |
|  | Congestive heart failure |
|  | Peripheral vascular disease |
|  | Cerebrovascular disease (Stroke) |
|  | Dementia |
|  | Chronic pulmonary disease (COPD) |
|  | Connective tissue disease |
|  | Peptic ulcer disease |
|  | Mild liver disease, without portal hypertension |
|  | Diabetes mellitus without end-organ damage |
| 2 |  |
|  | Hemiplegia or paraplegia |
|  | Moderate or severe renal disease |
|  | Diabetes mellitus with end-organ damage |
|  | Renal disease (CKD) |
|  | Any malignancy, including leukemia and lymphoma |
| 3 |  |
|  | Moderate or severe liver disease |
| 6 |  |
|  | Metastatic solid tumor |
|  | AIDS / HIV |

COPD, chronic obstructive pulmonary disease; CKD, chronic kidney disease; AIDS, acquired immunodeficiency syndrome; HIV, human immunodeficiency virus.

**Supplementary Table S2.** Carlson Comorbidity Index (CCI) scores evaluation from respondent’s comprehensive health status in enrolled participants of NHANES 2007-2018.

| Diseases | Score |
| --- | --- |
| Psoriasis | 0 |
| Hypertension | 0 |
| Hypercholesterolemia | 0 |
| Diabetes | 1 |
| Diabetic retinopathy | 2 |
| Hepatitis B | 1 |
| Hepatitis C | 1 |
| Kidney failure | 2 |
| Kidney stones | 1 |
| Asthma | 0 |
| Arthritis | 0 |
| Gout | 0 |
| Heart failure | 1 |
| Coronary heart disease | 0 |
| Heart disease | 0 |
| Stroke | 1 |
| Emphysema | 0 |
| Thyroid disease | 0 |
| Bladder cancer | 2 |
| Bone cancer | 2 |
| Brain cancer | 2 |
| Breast cancer | 2 |
| Cervical cancer | 2 |
| Colon cancer | 2 |
| Esophageal cancer | 2 |
| Gallbladder carcinoma | 2 |
| Kidney cancer | 2 |
| Tracheal carcinoma | 2 |
| Lukaemia | 2 |
| Liver cancer | 2 |
| Lung cancer | 2 |
| Lymphomas | 2 |
| Melanoma | 2 |
| Oral cancer | 2 |
| Nerver cancer | 2 |
| Ovarian cancer | 2 |
| Pancreatic cancer | 2 |
| Prostatic cancer | 2 |
| Rectal cancer | 2 |
| Skin cancer(non-melanoma) | 2 |
| Other skin cancer | 2 |
| Soft tissue cancer | 2 |
| Stomach cancer | 2 |
| Testicular cancer | 2 |
| Thyroid cancer | 2 |
| Endometrial cancer | 2 |
| Other cancers | 2 |
| COPD | 1 |
| Osteoporosis | 0 |

**Supplementary Table S3**. Predictors of depression among US adults

| Variable | Count part | | Logit part | |
| --- | --- | --- | --- | --- |
|  | RR (95% CI) | P-value | OR (95% CI) | P-value |
| CCI | 1.21(1.18,1.24) | <0.0001 | 1.25(1.21,1.29) | <0.0001 |
| Age | 0.99(0.98,0.99) | <0.0001 | 0.98(0.98,0.99) | <0.0001 |
| Sex |  |  |  |  |
| Female | ref | ref | ref | ref |
| Male | 0.62(0.57,0.68) | <0.0001 | 0.57(0.50,0.65) | <0.0001 |
| Race / Ethnicity |  |  |  |  |
| Non-Hisp. Black | ref | ref | ref | ref |
| Non-Hisp. White | 1.14(1.01,1.27) | 0.03 | 1.12(0.97,1.29) | 0.14 |
| Mexican American | 1.04(0.90,1.21) | 0.58 | 0.88(0.73,1.06) | 0.17 |
| Other Race | 1.30(1.14,1.49) | <0.0001 | 1.39(1.16,1.66) | <0.001 |
| BMI (kg/m^2^) | 1.02(1.01,1.02) | <0.0001 | 1.02(1.01,1.03) | <0.0001 |
| PIR | 0.80(0.77,0.82) | <0.0001 | 0.79(0.75,0.83) | <0.0001 |
| Marital status |  |  |  |  |
| Married, living with partner | ref | ref | ref | ref |
| Single, divorced, or widowed | 1.48(1.36,1.61) | <0.0001 | 1.60(1.42,1.79) | <0.0001 |
| Education |  |  |  |  |
| College or above | ref | ref | ref | ref |
| High school or below | 1.17(1.07,1.29) | <0.001 | 1.18(1.02,1.37) | 0.02 |
| Smoke |  |  |  |  |
| Never | ref | ref | ref | ref |
| Former | 1.28(1.14,1.43) | <0.0001 | 1.28(1.14,1.43) | <0.0001 |
| Now | 1.96(1.76,2.18) | <0.0001 | 1.96(1.76,2.18) | <0.0001 |
| Alcohol drinking |  |  |  |  |
| Never | ref | ref | ref | ref |
| Former | 1.39(1.19,1.62) | <0.0001 | 1.65(1.33,2.06) | <0.0001 |
| Mild | 1.13(0.97,1.31) | 0.12 | 1.25(1.03,1.51) | 0.02 |
| Moderate | 1.22(1.03,1.44) | 0.02 | 1.33(1.02,1.73) | 0.04 |
| Heavy | 1.22(1.04,1.43) | 0.02 | 1.28(1.02,1.61) | 0.04 |
| Physical activity |  |  |  |  |
| Active | ref | ref | ref | ref |
| Inactive | 1.70(1.49,1.94) | <0.0001 | 1.76(1.47,2.09) | <0.0001 |
| Hypertension |  |  |  |  |
| No | ref | ref | ref | ref |
| Yes | 1.19(1.08,1.31) | <0.001 | 1.34(1.15,1.58) | <0.001 |
| TC (mg/dL) | 1.00(1.00,1.00) | 0.01 | 1.00(1.00,1.00) | 0.02 |

Negative binomial (count part) and logistic regression (logit part) models under multivariable analysis for predictors of depression

**Supplementary Table S4**. Associations of CCI, and tertiles of CCI with depression in excluded adult participants with PHQ-9 test.

|  | N | Depression | Non-depression | Odds ratio (95% CI) |
| --- | --- | --- | --- | --- |
| CCI | 10840 | 623 | 5357 | 1.26 (1.21,1.31) |
| Tertiles of CCI |  |  |  |  |
| T1, CCI= 0 | 5883 | 187 | 2813 | ref |
| T2, CCI= 1 | 1980 | 144 | 1032 | 2.10 (1.67,2.64) |
| T3, CCI= [2,12] | 2977 | 292 | 1512 | 2.91 (2.40,3.53) |

The odds ratio (95% CI) was univariable logistic regression model. Only the population assessed for PHQ-9 testing was quantified and analyzed

**Supplementary Table S5**. Associations of CCI, tertiles of CCI, and CCI / depression group with mortality in excluded adult participants with follow up data.

|  | Alive | Death | Hazard ratio (95% CI) |
| --- | --- | --- | --- |
| CCI | 6615 | 769 | 1.41 (1.38,1.44） |
| Tertiles of CCI |  |  |  |
| T1, CCI= 0 | 5507 | 293 | ref |
| T2, CCI= 1 | 1677 | 287 | 3.3 (2.81,3.89) |
| T3, CCI= [2,12] | 2224 | 738 | 5.95 (5.20,6.81) |
| CCI / depression group |  |  |  |
| T1, depression (-) | 2676 | 93 | ref |
| T1, depression (+) | 172 | 14 | 2.21 (1.26, 3.88) |
| T2, depression (-) | 915 | 109 | 3.71 (2.81, 4.89) |
| T2, depression (+) | 125 | 16 | 3.62 (2.13, 6.15) |
| T3, depression (-) | 1209 | 296 | 6.92 (5.48, 8.74) |
| T3, depression (+) | 233 | 59 | 7.23 (5.22, 10.02) |

The hazard ratio (95% CI) was univariable Cox proportional hazard regression model. Only the population with follow-up data assumed alive or death was quantified and analyzed


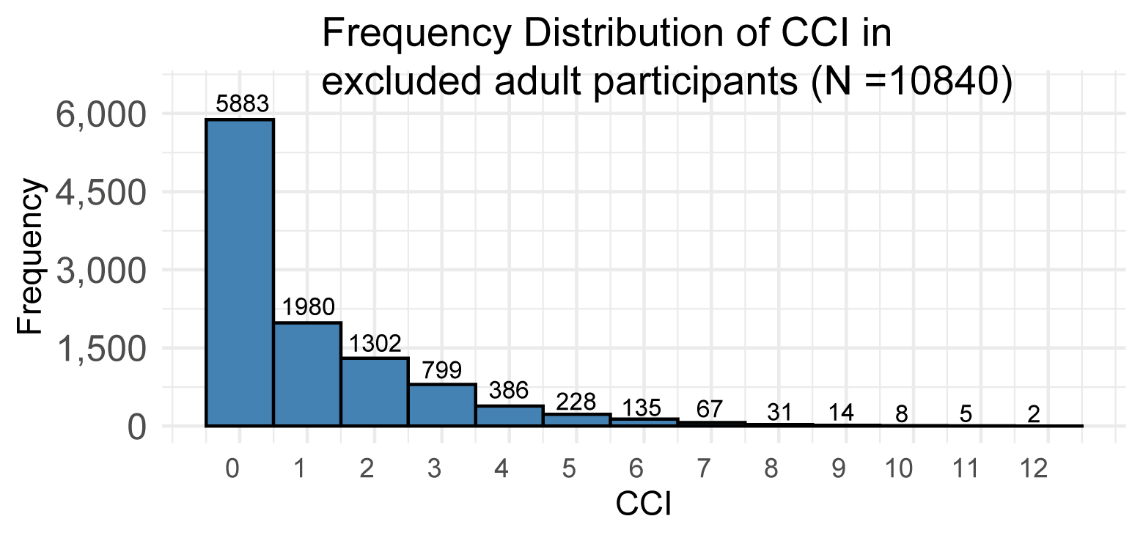


**Supplementary Figure S1.** Distribution of Carlson comorbidity index (CCI) in excluded adult participants with CCI evaluation (N =10840).
